# The effect of improved housing on indoor mosquito density and exposure to malaria in the rural community of Minkoameyos, Centre Region of Cameroon

**DOI:** 10.1186/s12936-020-03232-6

**Published:** 2020-05-03

**Authors:** Rachel L. Nguela, Jude D. Bigoga, Tedjou N. Armel, Tallah Esther, Dongmo Line, Njeambosay A. Boris, Tchouine Frederic, Riksum Kazi, Peter Williams, Wilfred F. Mbacham, Rose G. F. Leke

**Affiliations:** 1 Malaria Consortium-Cameroon Coalition Against Malaria (MC-CCAM), Bastos, PO Box 4256, Yaoundé, Cameroon; 2 Department of Public Health, Faculty of Medicine and Biomedical Sciences, University of Yaoundé I, Yaoundé, Cameroon; 3 National Reference Unit for Vector Control, The Biotechnology Centre, University of Yaoundé I, Yaoundé, Cameroon; 4 Department of Biochemistry, Faculty of Science, University of Yaoundé I, Yaoundé, Cameroon; 5 Architecture for Health in Vulnerable Environments (ARCHIVE Global), New York, USA

**Keywords:** Housing improvement, *Anopheles density*, *Malaria transmission*, Rural community, Cameroon

## Abstract

**Background:**

This study evaluated the effectiveness of improved housing on indoor residual mosquito density and exposure to infected Anophelines in Minkoameyos, a rural community in southern forested Cameroon.

**Methods:**

Following the identification of housing factors affecting malaria prevalence in 2013, 218 houses were improved by screening the doors and windows, installing plywood ceilings on open eaves and closing holes on walls and doors. Monthly entomological surveys were conducted in a sample of 21 improved and 21 non-improved houses from November 2014 to October 2015. Mosquitoes sampled from night collections on human volunteers were identified morphologically and their parity status determined. Mosquito infectivity was verified through *Plasmodium falciparum* CSP ELISA and the average entomological inoculation rates determined. A Reduction Factor (RF), defined as the ratio of the values for mosquitoes collected outdoor to those collected indoor was calculated in improved houses (RFI) and non-improved houses (RFN). An Intervention Effect (IE = RFI/RFN) measured the true effect of the intervention. Chi square test was used to determine variable significance. The threshold for statistical significance was set at P < 0.05.

**Results:**

A total of 1113 mosquitoes were collected comprising *Anopheles* sp (58.6%), *Culex* sp (36.4%), *Aedes* sp (2.5%), *Mansonia* sp (2.4%) and *Coquillettidia* sp (0.2%). Amongst the Anophelines were *Anopheles gambiae* sensu lato (*s.l*.) (95.2%), *Anopheles funestus* (2.9%), *Anopheles ziemanni* (0.2%), *Anopheles brohieri* (1.2%) and *Anopheles paludis* (0.5%). *Anopheles gambiae* sensu stricto (*s.s.*) was the only *An. gambiae* sibling species found. The intervention reduced the indoor *Anopheles* density by 1.8-fold (RFI = 3.99; RFN = 2.21; *P *= *0.001*). The indoor density of parous *Anopheles* was reduced by 1.7-fold (RFI = 3.99; RFN = 2.21; *P *= *0.04*) and that of infected *Anopheles* by 1.8-fold (RFI = 3.26; RFN = 1.78; *P *= *0.04*). Indoor peak biting rates were observed between 02 a.m. to 04 a.m. in non-improved houses and from 02 a.m. to 06 a.m. in improved houses.

**Conclusion:**

Housing improvement contributed to reducing indoor residual anopheline density and malaria transmission. This highlights the need for policy specialists to further evaluate and promote aspects of house design as a complementary control tool that could reduce indoor human–vector contact and malaria transmission in similar epidemiological settings.

## Background

Malaria threatened nearly half of the world’s population in 2012 [1], with a total of 198 million cases reported in 2013 [2]. Malaria is endemic in 43 sub-Saharan Africa countries, where it constitutes the leading cause for outpatient consultations and hospitalization [1]. In Cameroon in 2013, malaria accounted for 28.7% of all consultations in health facilities, 49.8% of hospitalizations, 22% of deaths across all age groups, and 45% of deaths amongst children less than 5 years [3]. Despite increasing efforts expended by the Cameroon government to control the disease, the endemicity is seemingly stagnant across most parts of the country, and is highly heterogeneous across the various geo-ecological and climatic settings. Children less than 5 years old and pregnant women are the most affected [4]. The intensity and duration of malaria transmission is greatly influenced by climate and geography. In many endemic biotopes, the situation is further worsened by increased drug resistance in *Plasmodium falciparum*, the prevailing parasite species, inconsistent allocation and inadequate use of vector control measures, the occurrence of a vast plethora of permissive and efficient vectors of *P. falciparum* [5, 6], and the occurrence and spread of insecticide resistance in the major vectors [7, 8]. Of the 52 Anopheles species described so far in Cameroon, 17 have been reported to support the development and propagation of malaria parasites, amongst which are six major species (*Anopheles gambiae, Anopheles coluzzii, Anopheles arabiensis, Anopheles funestus, Anopheles nili* and *Anopheles moucheti*). The rest play only minor, secondary roles in transmission locally.

Although vector control is fundamentally the most successful strategy for malaria prevention and control in Cameroon (and beyond), its effectiveness over the years has relied essentially on the use of long-lasting insecticidal nets (LLIN). Meanwhile the application of indoor residual spraying (IRS) is currently being considered in the country [9]. Nevertheless, the evolution and spread of insecticide resistance, especially to pyrethroids and changes in vector behaviour in the presence of these interventions are major threats to their efficacy [10–17]. The Cameroon Multiple Indicator Demographic Health Survey conducted in 2011 revealed low LLIN coverage in households and increase non compliance with user practices as some of the reasons for the country’s limited progress in malaria control [4]. This emphasizes the need to develop new and alternative or complementary strategies for effective malaria vector control in line with the recommendations of the World Health Organization (WHO) [5].

Housing is increasingly being recognized as an important determinant of health outcomes [18]. Several studies have demonstrated the relationship between housing design and global health issues, such as parasitic diseases and flooring material, respiratory diseases and indoor ventilation, vector-borne diseases and screening of openings. History in Europe portrays the potential for housing improvements as a legitimate strategy to effectively contribute towards malaria elimination [19, 20]. Housing improvements through screening of windows and doors, closing of eaves and crevices, patching of walls and roofs could help reduce malaria transmission [21–26].

In many African countries, the biting and feeding activity of the main malaria vectors tend to increase at night when humans are mainly indoors [21–30]. Houses with openings at the level of eaves, walls, windows, doors and/or ceilings will enhance mosquito entry, exposing its occupants to higher risks of malaria [31–34]. Earlier studies in Cameroon revealed higher malaria parasite prevalence and density amongst individuals living in poorly constructed houses (wooden plank houses) compared to those in cement and brick houses [7].

Typical housing in many areas has openings on the eaves, walls, windows and doors. These are important determinants that could facilitate mosquito entry, thus increasing human vector contact and exposure to infective bites. This study, therefore, aimed to determine the effectiveness of improved housing on indoor residual mosquito density and exposure to malaria-carrying Anophelines in the rural endemic community of Minkoameyos in the Centre region of Cameroon.

## Methods

### Study area

The study was carried out in Minkoameyos, a locality situated in the Nkol-Nkoumou health area of the Nkolbisson health district. It is located 25 km to the west of Yaoundé, the capital city of Cameroon. This village is about 731 m above the sea level at latitude 11, 42° North and longitude 3, 87° East. The climate is of the Guinean Equatorial type with two dry seasons (July to August and November to February) and two rainy seasons (March to June and September to November) [35]. The annual rainfall and temperature averages 1650 mm and 24 °C, respectively, with a relative humidity less than 80% [36]. It is a rural community harbouring approximately 710 households with an average of seven people (including two children less than 5 years) old per household. The local ethnic groups comprise mainly of the indigenous Ewondos, and some members of the Bassa, Bamileke, Bamoun and Eton tribes. They depend essentially on farming and small scale businesses for subsistence. Minkoameyos is in the south Cameroonian Equatorial forest strata, where malaria transmission is known to occur perennially with *An. gambiae* sensu lato (*s.l*.) being the major vector species, and *P. falciparum* as the predominant parasite species [37].

### Study design and housing modifications

**Fig. 1:**
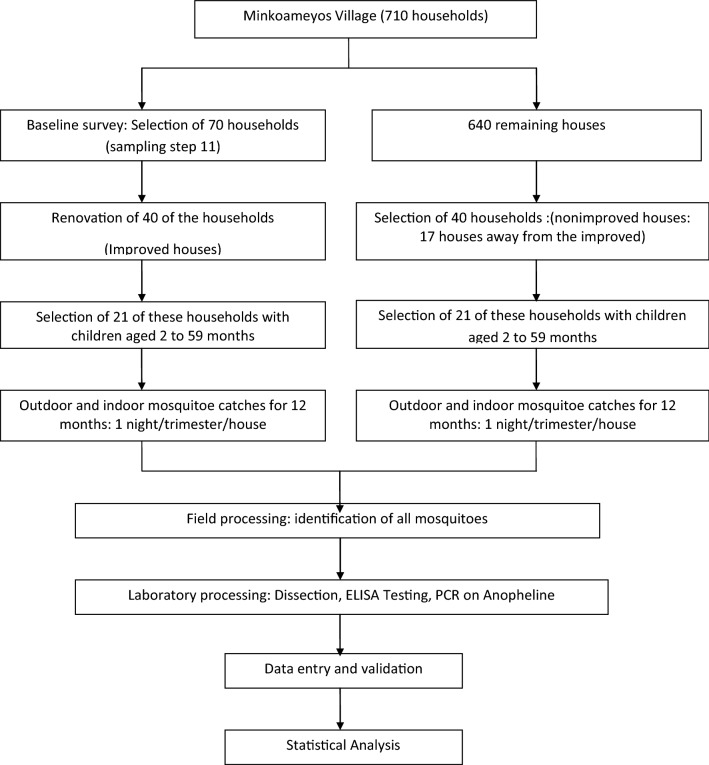
Sampling and data collection process for the entomologic study

This was a longitudinal entomological study that lasted 12 months, from October 2014 to November 2015. The main intervention was housing modification targeting windows, doors, eaves, walls and roof to limit mosquito access into houses. For the control arm of the intervention, none of the above was done on the assigned houses. As described in Fig. 1, the selection of households for the study was through a systematic random sampling. Outcome parameters were the entomological indices of malaria transmission. Prospective mosquito collection was done in both the intervention and control houses. Measures of the entomological indices for malaria transmission in the two groups were compared for effectiveness.

**Fig. 2:**
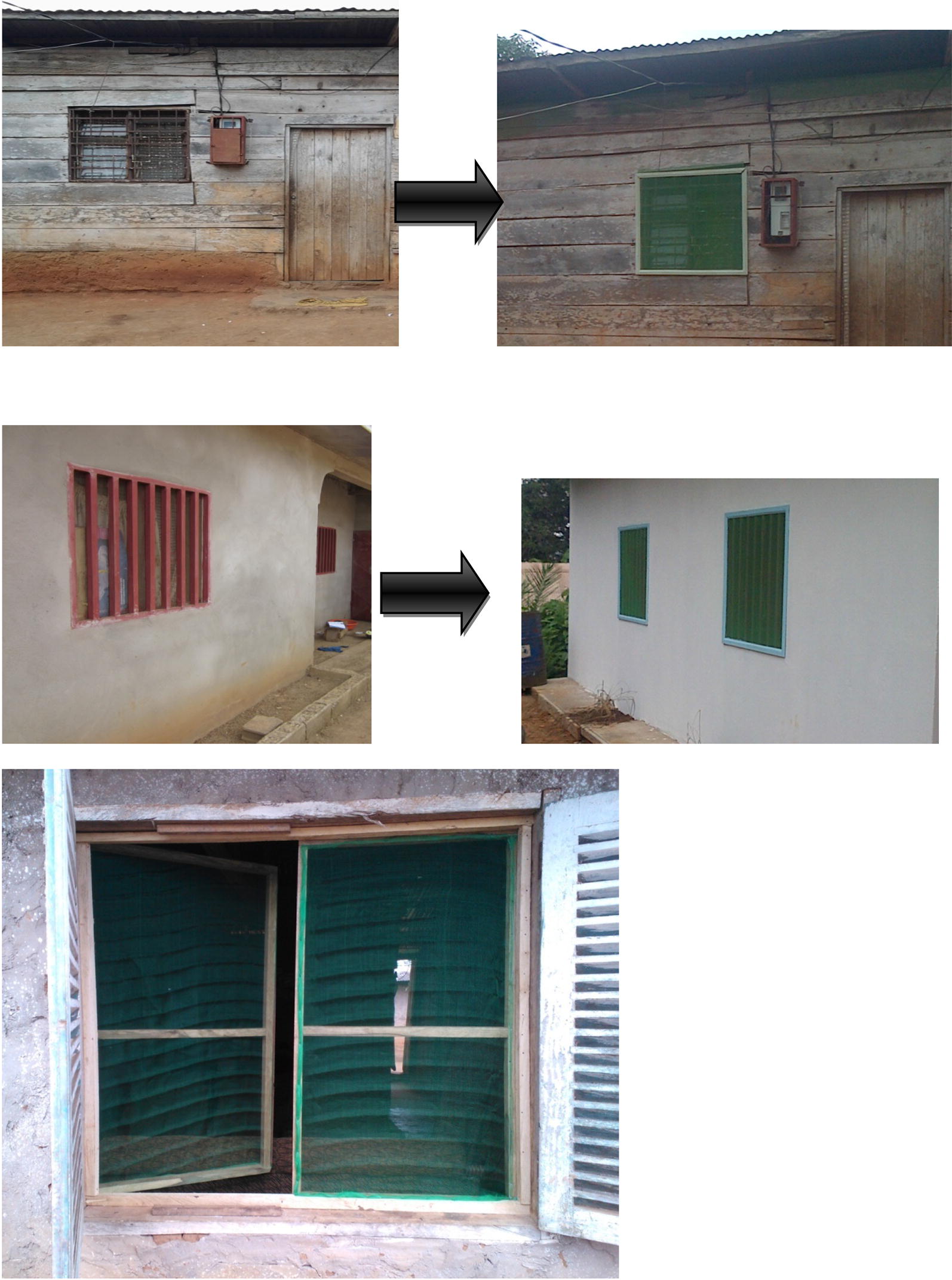
Improvement on windows

**Fig. 3:**
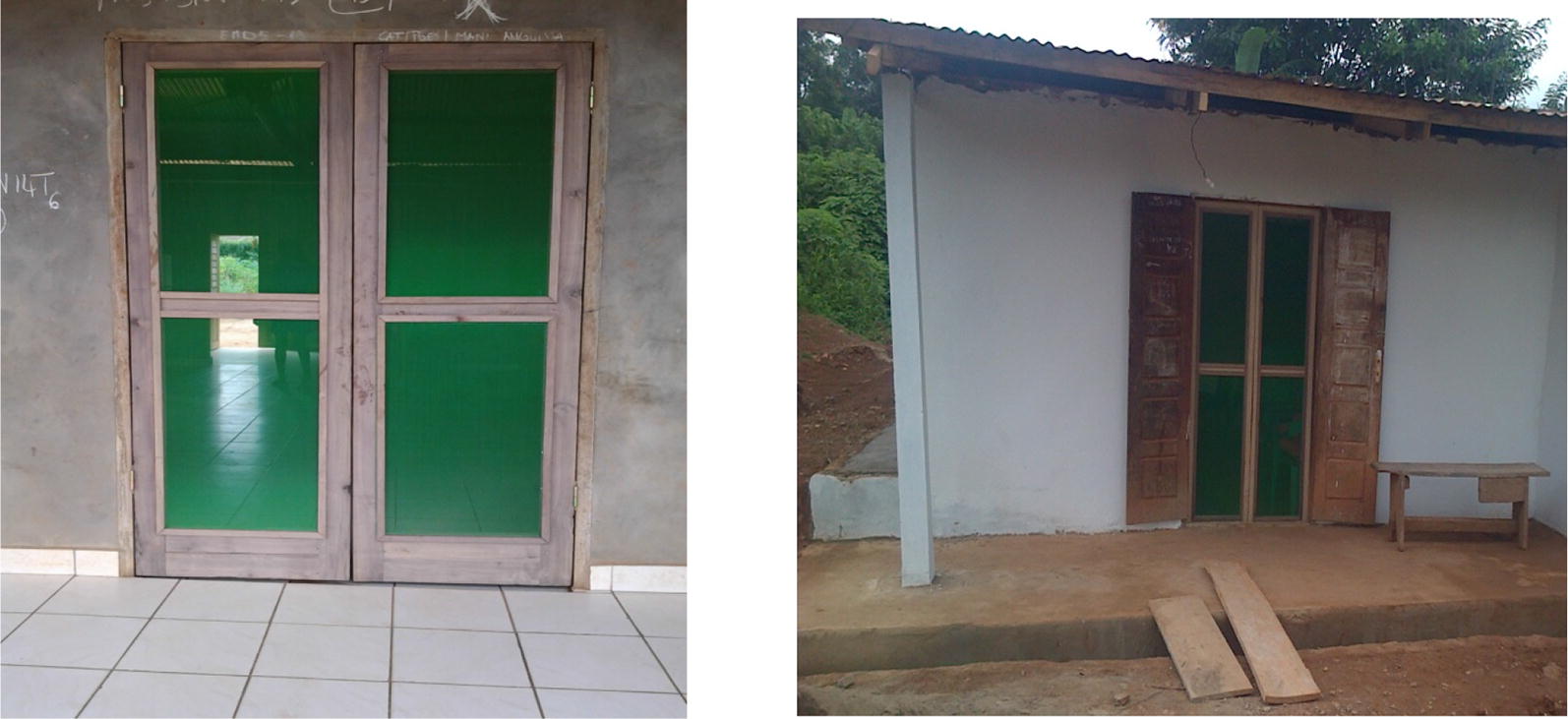
Improvement on doors

**Fig. 4:**
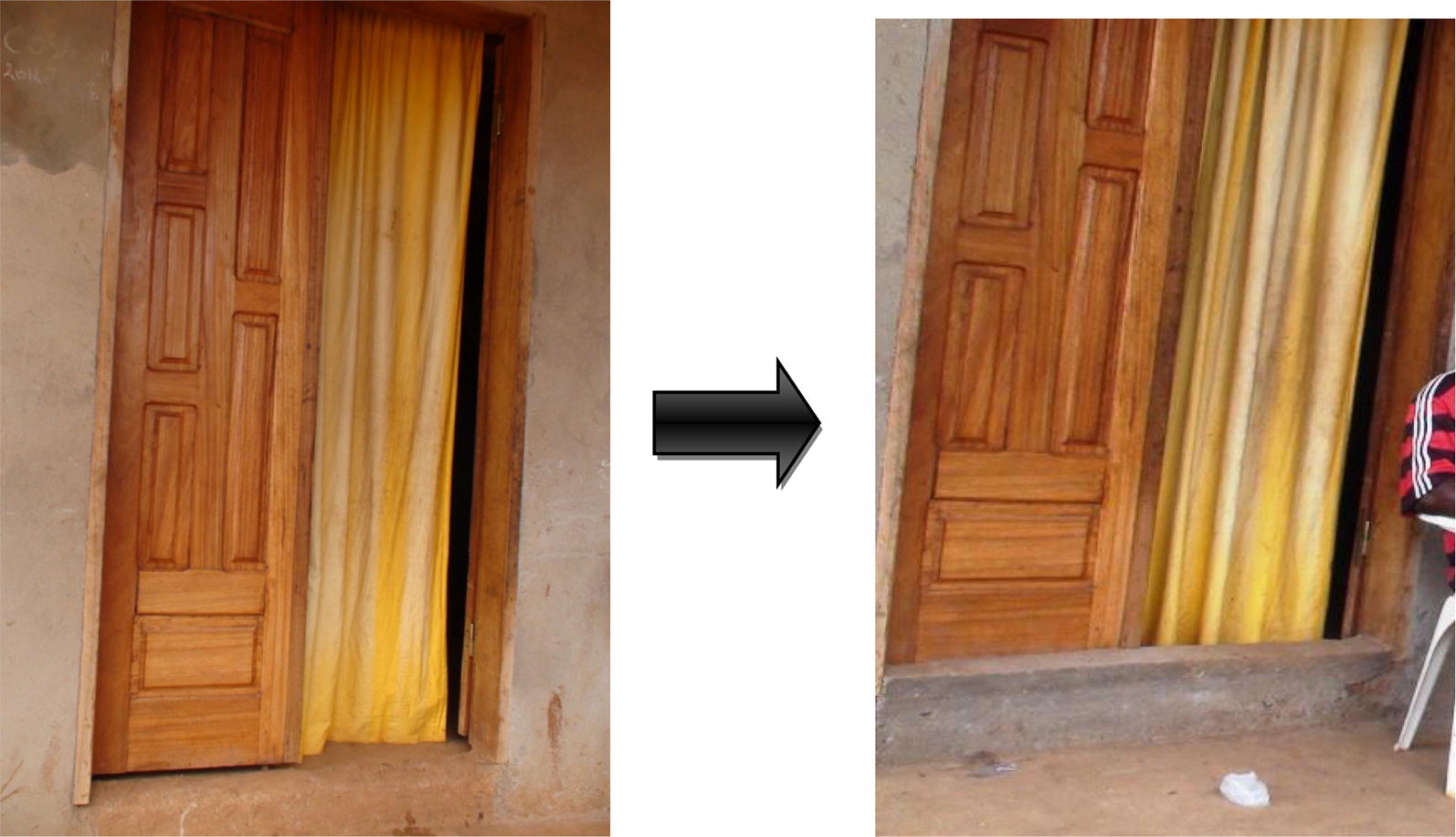
Improvement on door steps

**Fig. 5:**
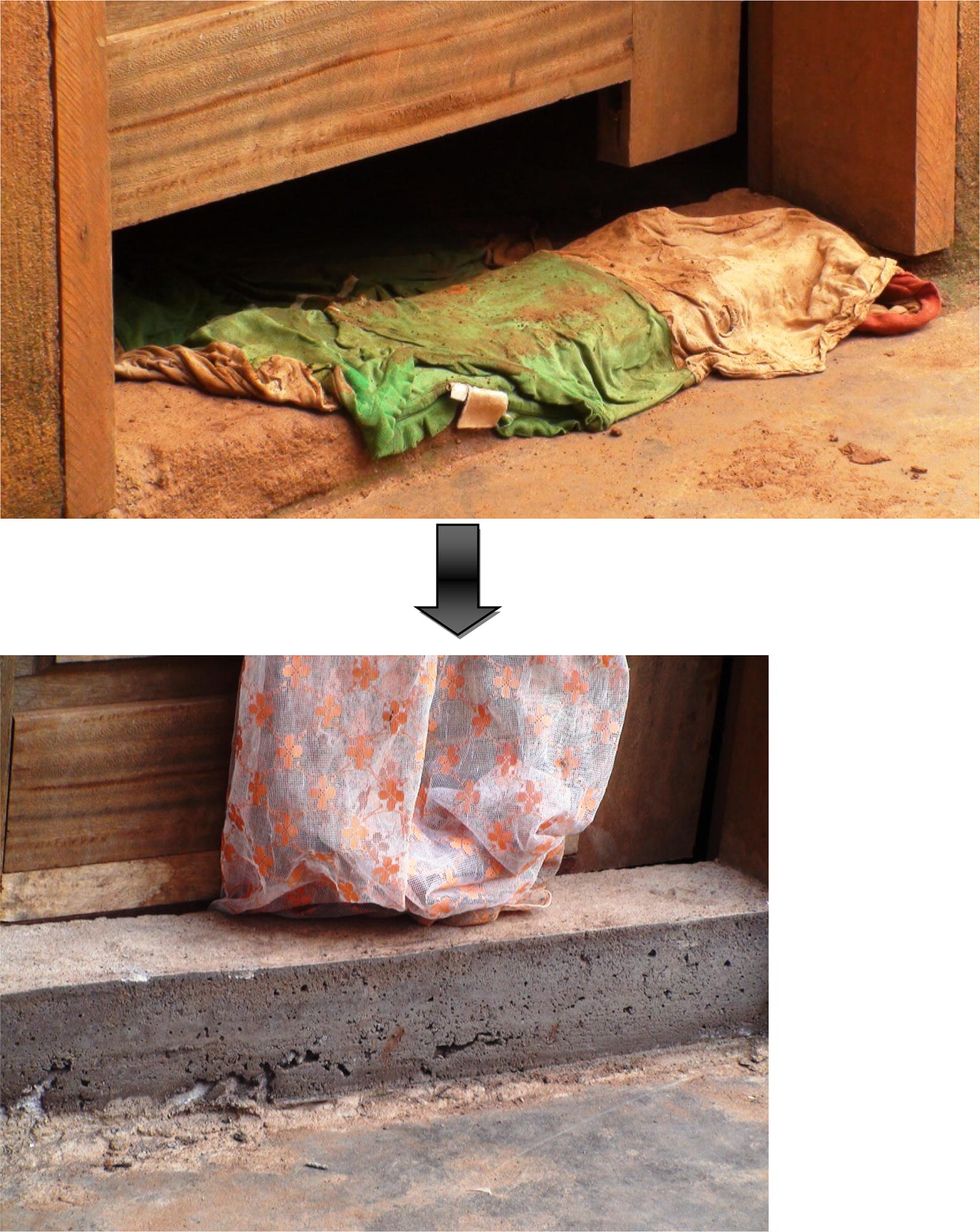
Improvement on doors

**Fig. 6:**
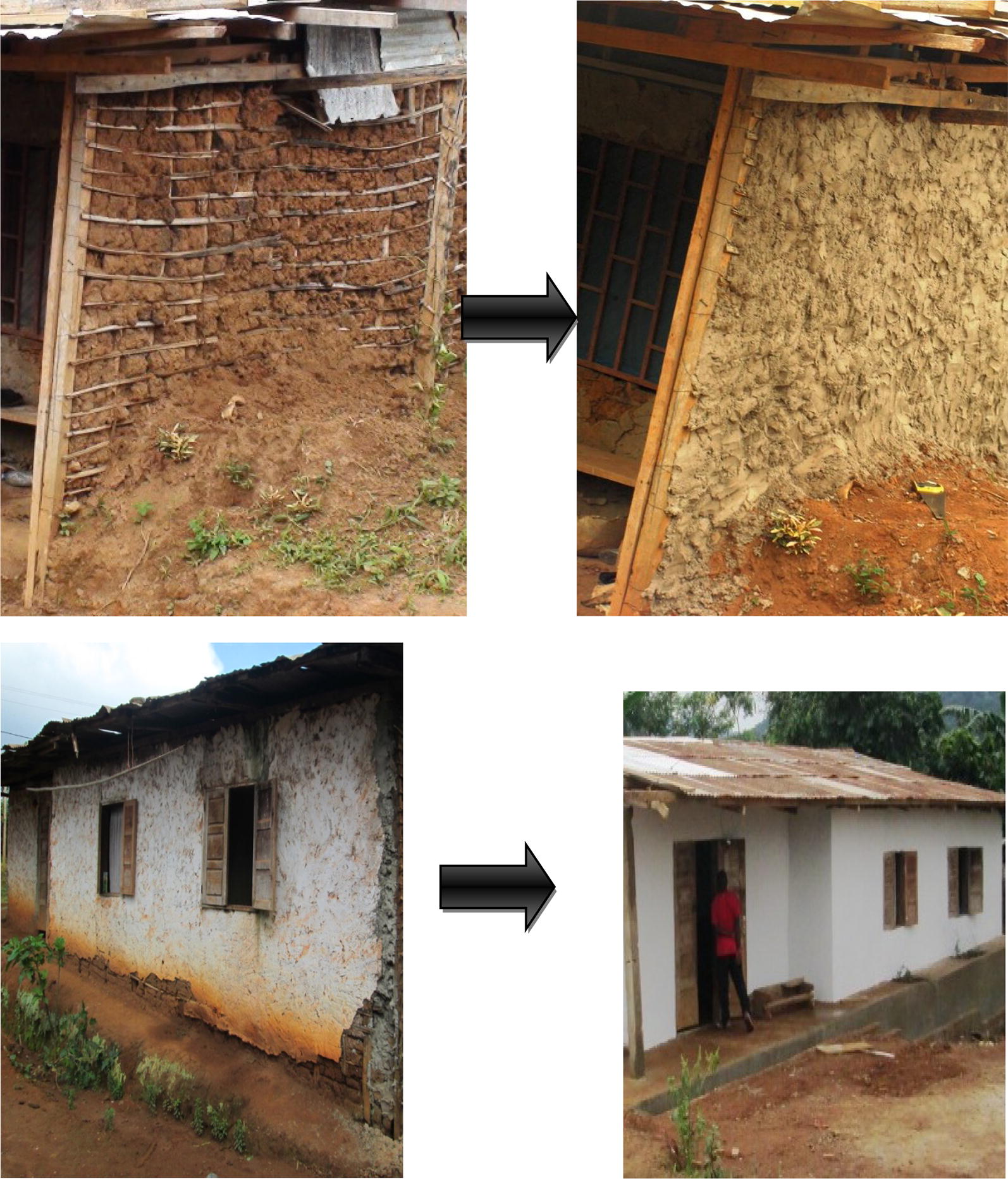
Improvement on walls

**Fig. 7:**
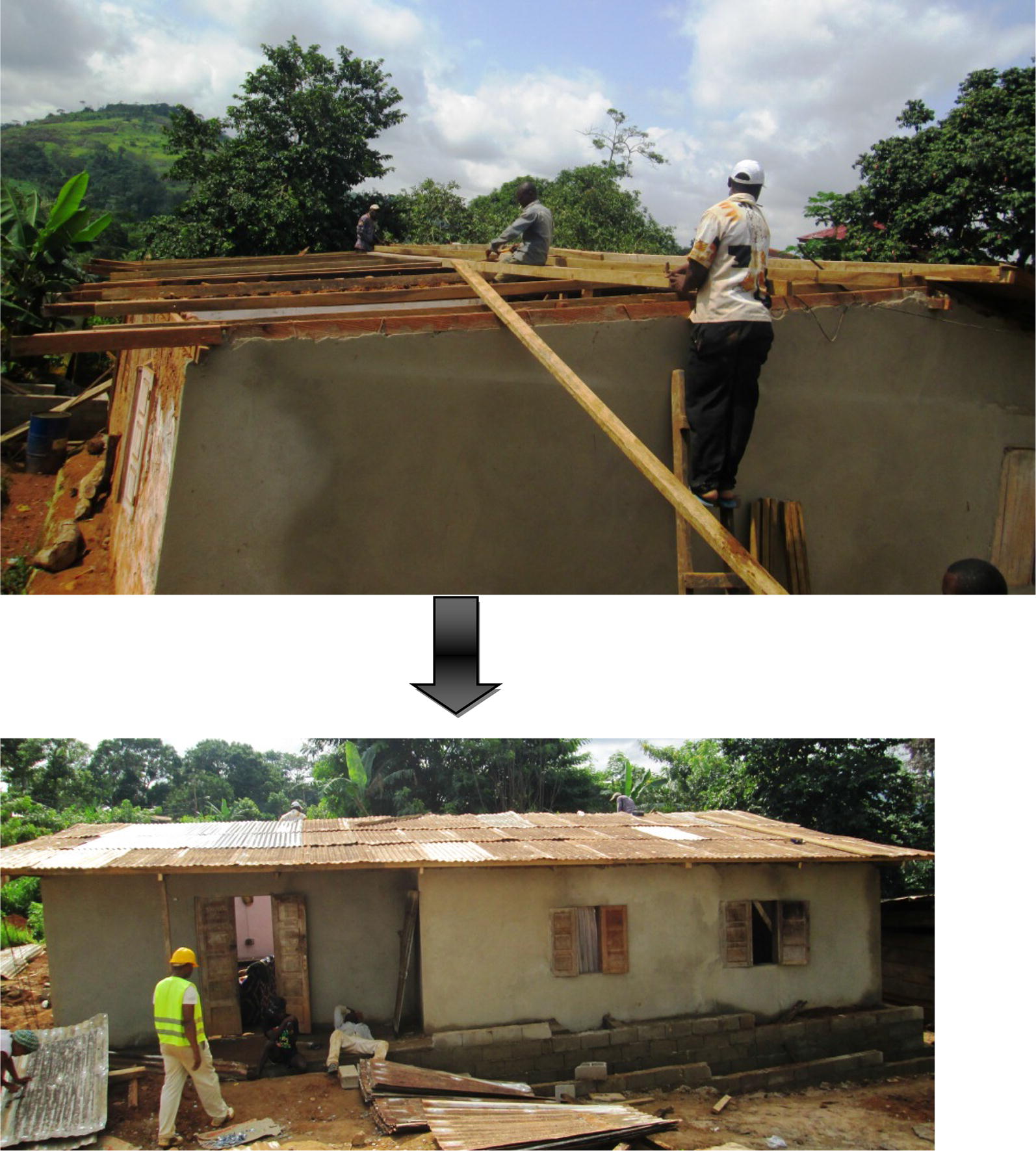
Improvement on roofs

Specifically, in intervention houses, screened doors with metallic netting and wooden frame were fabricated and installed on all existing doors leading to outside. For houses with windows opening towards outside, a second window with metallic netting and wooden frame was made and mounted on the existing window frames. Where the windows were hanging inside the house when opened, or could be opened without hanging at all, a piece of metallic netting was adapted to the outer parts of the window frame, using wooden cover joints. Sheets of compacted wood were used to block all opened eaves. All holes on the roofs and walls were closed using same type of material used during initial construction by the house owners. Figures 2, 3, 4, 5, 6 and 7 show some of the improvements that were done on the house structures.

For the control arm of the intervention, no housing modification was done during the study period. Both study arms were found in the same community. They had the same source of information regarding malaria prevention and sought the same health facilities for malaria case management.

### Field collection and processing of adult Anophelines

Every month, during two consecutive nights, mosquitoes were sampled from 06:00 p.m. to 06:00 a.m. from improved and non-improved houses using the human landing catch (HLC) method. Mosquitoes were collected indoors and outdoors in three randomly selected houses (at least 50 m apart) each night, with rotation between houses at different locations in order to cover every section of the village. A team of four trained volunteers (two during the first half on the night and the others during the second half of the night) collected mosquitoes in each house. Only adequately trained volunteers were allowed to collect mosquitoes. In each selected house, one collector sat inside the house (indoor) and the other on the veranda (outdoor) where they collected mosquitoes as soon as they landed on their exposed lower limbs. In order to avoid bias due to differential attractiveness, the two volunteers swapped locations (indoor and outdoor) every 2 h during night and during which time an entomologist visited the teams and collected of mosquitoes. These mosquitoes were sorted by genus and the anophelines identified morphologically using keys of Gillies and De Meillon (1947), and Gillies and Coetzee (1987) [38, 39]. The ovaries of all unfed females were dissected for parity status determination as described by Detinova et al. [40]. All dissected and undissected mosquitoes were individually stored desiccated in tubes for subsequent laboratory analyses.

### Laboratory processing of Anophelines

A proportion of the collections belonging to the *An. gambiae* complex was further identified to species level using molecular assays. Genomic DNA of each individual specimen was extracted using DNAzol protocol [41] and PCR amplified to determine species according to Favia et al. [42]. The head and thorax portions of each female *Anopheles* collected were separated from the rest of the body, homogenized in grinding buffer (0.5% Casein, 0.1 N NaOH) and used to check for the presence of *P. falciparum* circumsporozoite protein (CSP) by enzyme-linked-immunosorbent assay (ELISA) [43, 44]. For each species, the infection rate was calculated and the entomological inoculation rate determined. To minimize false positive CSP ELISA, only high absorbance readings were considered (mean plus three standard deviations of the negative controls).

### Data analysis

For each house, information on each mosquito collected from field and laboratory procedures during each night catch was recorded using a questionnaire. The data were entered into Epi Info^™^ software by two trained data clerks and both databases then sorted and cleaned for statistical analysis. Man biting rate (ma) was calculated as the average number of bites received from *Anopheles* species per person each night of collection. Infection rate (IR) was calculated as the proportion of *Anopheles* species tested positive for *P. falciparum* CSP by ELISA. The entomological inoculation rate (EIR) was determined as the product of the infection rate (IR) and the man biting rate (ma). A Chi square test was used to determine variable significance. The threshold for statistical significance was set at P < 0.05.

With regards to measures of the intervention effect, the following parameters were considered:

- Reduction Factor (RF) given as the ratio of the values for mosquitoes collected outdoor to those collected indoor. (RFI = Reduction Factor in improved houses and RFN = Reduction Factor in non-improved houses);
  Where RFI > 1, intervention had a reduction effect between outdoor and indoor on a specific entomologic index;
  RFI < 1, intervention had an exposing effect between outdoor and indoor on a specific entomological index;
- The Intervention Effect (IE) = Measure of the true effect of the intervention in the population.
  IE = RFI/RFN
  Where IE > 1, meant the intervention has protective effect in the overall population (on the entomological index of interest);
  Where IE < 1 meant the intervention is non-protective in the overall population (on the entomological index of interest).

## Results

### Mosquito composition and density

As shown in Table 1, a total of 1105 mosquitoes were collected comprising 647 (58.6%) *Anopheles* sp., 402 (36.4%) *Culex* sp., 28 (2.5%) *Aedes* sp. and 2 (0.2%) *Coquellitidia* sp. The Anophelines comprised of *An. gambiae s.l.* (95.2%), *Anopheles funestus* (2.9%), *Anopheles brohieri* (1.2%), *Anopheles paludis* (0.5%) and *Anopheles ziemanni* (0.2%). Of the 647 Anophelines, 154 (23.8%) collected indoors comprising *An. gambiae s.l.* 149 (96.7%), *An. funestus* 4 (2.6%), and *An. ziemanni*1 (0.6%). 493 (76.2%) were collected outdoor, made up of *An. gambiae s.l.* 467 (94.7%), *An. funestus* 15 (3.04%), *An. paludis* 3 (0.6%) and *An. brohieri* 81.6 (%). *An. gambiae* sensu stricto (*s.s*.) was the only member of the *An. gambiae* complex found.

**Table 1:** Malaria transmission indices in Minkoameyos

Entomological index		*Anopheles* species					
		*An. brohieri*	*An. funestus*	*An. gambiae*	*An. ziemanni*	*An. paludis*	Total
							*Anopheles*
Composition							
n		8	19	616	1	3	647
%		1.2	2.9	95.2	0.2	0.5	100
Man biting rate (b/p/n)		0.001	0.003	0.094	0	0	0.098
Parous/dissected		1/8	7/13	290/465	1/1	0/1	299/488
Parity rate							
%		12.5	53.8	62.4	100	0	61.3
(95% CI)		(0.3–52.7)	(25.1–80.8)	(57.8–66.8)	n/a	n/a	(56.8–65.6)
Tested for CSP		8	19	584	1	3	615
CSP rate							
%		0	52.6	33.7	100	100	34.2
(95% CI)		n/a	(28.9–75.6)	(29.9–37.7)	n/a	n/a	(30.4–38.1)
EIR (ib/p/n)							
%			0.01	0.28	0	0	0.29

*n/a* Not applicable, *EIR* Entomological inoculation rate, *CSP* circumsporozoite protein, *ib/p/n* infective bites per person per night

With regards to the proportion of *Anophelines* collected based on housing status (improved/non-improved), 429 were collected from improved houses, of which 20.04% were indoor and 79.96% outdoor. In the non-improved houses, 218 *Anophelines* were collected, amongst which 31.2% was indoor and 68.8% outdoor.

### Nocturnal activity and biting cycle of anophelines

**Fig. 8:**
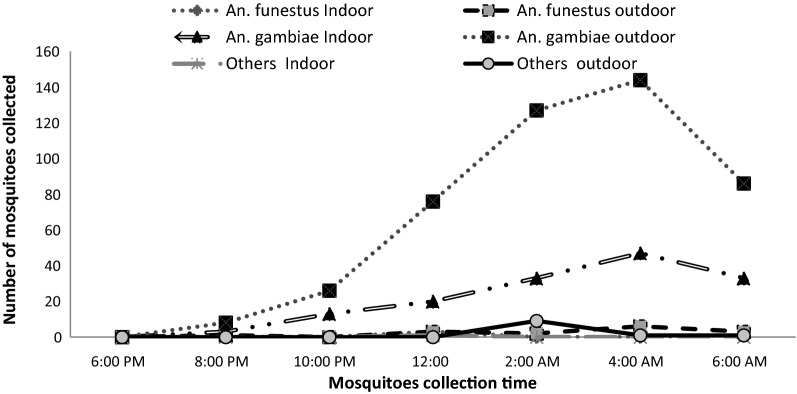
Indoor and outdoor biting cycles of Anopheles species in Minkoameyos

Overall, the average man biting rate was observed to increase gradually between 6 p.m. and 4 a.m., peaking between 2 a.m. and 4 a.m. and then slowly declining to 6 a.m. (Fig. 8). The overall man biting rate for the *Anopheles* was 0.098 bites per person per night (b/p/n). *Anopheles gambiae* was the most aggressive species, representing 95.2% of the total number of bites (0.094b/p/n) with peak biting hours between 2 a.m. and 4 a.m. regardless of the place of bite. Despite the small number collected compared to *An. gambiae*, the peak biting hours for *An. funestus,* was also observed at the same period both indoor and outdoor (Fig. 8).

### Parity rates

A total of 488 female *Anopheles* was dissected for parity status with an overall parity rate of 61.3% (Table 1). Segregating by species, the parity rates were 62.4% (290/465), 53.8% (7/13), 100% (1/1) and 12.5% (1/8) for *An. gambiae, An. funestus, An. ziemanni* and *An. brohieri,* respectively.

### Infection rates and entomological inoculation rates

A total of 615 female *Anopheles* mosquitoes were processed to ascertain the presence of *P. falciparum* circumsporozoite protein by CSP-ELISA. Of these, 210 were infected, giving an overall circumsporozoite protein rate of 34.2% (Table 1). Despite The circumsporozoite protein rate for *An. gambiae* (33.6%), the most abundant species, was lower compared to *An.* funestus (52.6%). While the lone *An. ziemanni* and 3 *An. paludis* collected were infected, none of the *An. brohieri* captured was infected. The intervention did not reduce indoor sporozoite infection rates of all *Anopheles* (IE = 1.1). It however reduced relative indoor sporozoite infection rates of *An. gambiae* by 1.8-fold. The overall average EIR was 0.29 infective bites per person per night (ib/p/n) with *An. gambiae* and *An. funestus* contributing to most of the transmission (Table 1).

### Effect of house improvement on entomological indices

#### Effect of house improvement on mosquito density

In the improved homes, the relative number of indoor *Anopheles* significantly reduced by 1.8-fold (RFI = 3.99; RFN = 2.21; P = 0.001) compared to the unimproved houses. In those improved homes, the relative number of *An. gambiae* entering houses increased by 1.7-fold (RFI = 3.81; RFN = 2.26; P = 0.004). Although the number of *An. funestus* collected indoors was 12-fold lower than the number collected outdoors in the improved houses, this effect did not differ statistically (RFI = 12; RFN = 1; P = 0.07) probably due to the small sample size (Table 2).

**Table 2:** Effect of housing improvement on malaria transmission indices in Minkoameyos

Mosquito species	Improved houses				Unimproved houses				IE	P-value
	Number of *Anopheles*			RFI	Number of *Anopheles*			RFN		
	Indoor	Outdoor	Total		Indoor	Outdoor	Total			
*An. funestus*	1	12	13	12	3	3	6	1	12	0.07
*An. gambiae*	84	320	404	3.81	65	147	212	2.26	1.7	0.004
Other *Anopheles*	1	11	12	11	0	0	0	0	n/a	n/a
All *Anopheles*	86	343	429	3.99	68	150	218	2.21	1.8	0.001

*n/a* Not applicable, *RFI* Reduction factor in improved houses, *RFN* Reduction factor in non-improved houses, *IE* Intervention effect

### Effect of house improvement on mosquito parity status

Table 3 summarizes the effect of the intervention on the number of parous anophelines by species. Improving houses generally led to a reduction in the number of parous anophelines collected indoor by 1.7-fold (RFI = 4.48; RFN = 2.67; P = 0.05). The relative number of parous *An. gambiae* significantly reduced by 1.8-fold (RFI = 4.32, RFN = 2.63; P = 0.03). The intervention was associated with 1.3-fold reduction in indoor parous rates for *An. gambiae* and 1.2-fold overall reduction of indoor parous rates (Table 3).

**Table 3:** Effect of housing improvement on parity rate of Anopheles population

Mosquito specy	Factor	Improved houses				Unimproved houses				IE	P-value
		Indoor	Outdoor	Total	RFI	Indoor	Outdoor	Total	RFN		
All *Anopheles*	Parous (n)	31	139	170	4.48	35	94	129	2.67	1.7	0.05
	Parity rate (%)	43.1	59.6	55.74	1.39	62.5	74.02	70.49	1.18	1.2	n/a
*An. funestus*	Parous (n)	1	4	5	4	0	2	2	n/a	n/a	n/a
	Parity rate (%)	100	66.6	71.43	0.67	0	66.6	33.33	n/a	n/a	n/a
*An. gambiae*	Parous (n)	29	134	163	4.62	35	92	127	2.63	1.8	0.03
	Parity rate (%)	41.4	61.4	56.6	1.48	66.04	74.2	71.75	1.12	1.3	n/a
Other anopheles	Parous (n)	1	1	2	1	0	0	0	n/a	n/a	n/a
	Parity rate (%)	100	11.1	20	0.11	n/a	n/a	n/a	n/a	n/a	n/a

*n/a* Not applicable, *RFI* Reduction factor in improved houses, *RFN* Reduction factor in non-improved houses, *IE* Intervention effect

### Effect of house improvement on entomological inoculation rate

Table 4 depicts the indoor and outdoor variation in entomological inoculation rates (EIR) in the two groups of houses. It was observed that improving the houses led to a reduction in the number of infective bites received per person per night indoors. A relative reduction of 1.7-fold (RFI = 4.84, RFN = 2.81) for all *Anopheles* and 1.6-fold (RFI = 4.75; RFN = 3.04) for *An. gambiae* was recorded.

**Table 4:** Effect of housing improvement on indoor and outdoor EIR of Anopheles

Mosquito species	Factor	Improved houses				Unimproved houses				IE	P-value
		Indoor	Outdoor	Total	RFI	Indoor	Outdoor	Total	RFN		
All *Anopheles*	EIR (ib/p/n)	1.67	8.07	0.048	4.84	1.24	3.49	2.34	2.81	1.7	n/a
*An. funestus*	EIR (%)	0	75	69.23	0	33.33	0	17	n/a	n/a	n/a
*An. gambiae*	EIR (ib/p/n)	1.64	7.77	0.047	4.75	1.13	3.43	2.24	3.04	1.6	n/a
Other *Anopheles*	EIR (%)	0.007	0.48	0.001	60.5	n/a	n/a	n/a	n/a	n/a	n/a

*n/a* Not applicable, *b/p/n* Bites per person per night, *EIR* Entomological Inoculation rate

### Effect of house improvement on the night biting cycle of the *anopheles*

**Fig. 9:**
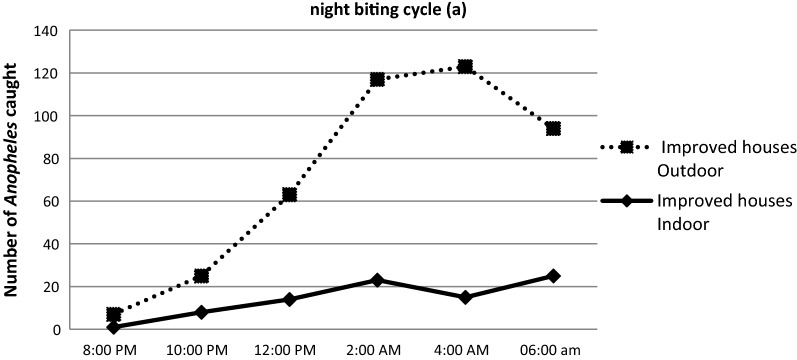
Night biting cycle of the anopheles in improved houses

**Fig. 10:**
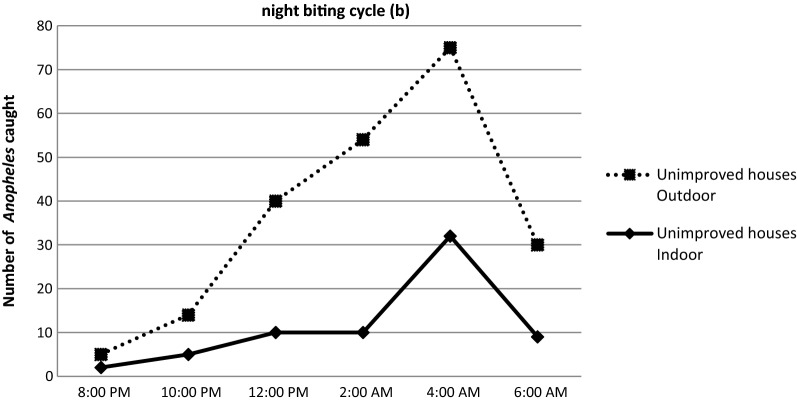
Night biting cycle of the anopheles in the Unimproved houses

As described in Fig. 9, the number of mosquitoes caught indoor in improved houses rose from 8 p.m. to 2 a.m. A reduction was subsequently observed up till 4 a.m. At this point a second increase started. At 6 a.m., when the catches were stopped, the highest number of mosquitoes collected indoor was found. Indoor and outdoor *Anopheles* abundance displayed the same hourly variation, with a peak at 4:00 a.m. (Fig. 10) in non-improved houses. In both group of houses, mosquitoes continued biting at 6:00 a.m. when the catches were stopped.

## Discussion

### Entomological indices

*Anopheles gambiae s.s.* and *An. funestus* were the main *Anopheles* species collected, followed by *An. ziemanni* and *An. paludis,* with an appearance of *An. brohieri.* During this study, most of the *Culicine* species collected was *Culex.* Its presence might have been due to the proximity of the study site to the city of Yaoundé, from where polluted water frequently drains, especially in run-offs to accumulate in this area, creating prolific breeding sites for this species. This high density constitutes an important source of nuisance by these mosquitoes in the community [45, 46], and an important risk factor for the diseases vectored by this species.

*Anopheles gambiae* as reported previously [46] was the most aggressive species found. Peak biting hours were consistently observed between 2 a.m. and 4 a.m. regardless of the place of bite (indoor or outdoor). This biting period showed some deviation from the prototype described previously by Gillies and De Meillon, which is from 10 p.m. to 2 a.m. in most endemic areas of Africa [47]. This highlights the ability of *Anopheles* species to frequently change and adapt their biting and feeding habits locally, as well as the need for designing control strategies that would be readily amenable locally. The high man biting rates could have also been due to policy gaps in the intervention strategies and non-compliance with LLIN use. At the time of this study, the LLIN mass distribution campaign had happen more than 4 years ago in the community. The LLIN in households were already older than 4 years and might have significantly lost their efficacy.

The high parous rate observed especially among the major vector species, *An.* g*ambiae s.s*., is indicative of the gradual accumulation of ageing adult population over time in this area. This is epidemiologically dangerous, as the mosquitoes will be able to survive long enough with increased chances of multiple feeding on humans and being able to transmit malaria over and again. In addition, it might be that the anti-vector measures in place are in adequate or not efficacious or that the vectors might have simply developed strategies to circumvent the intervention and survive beyond the intrinsic incubation period of the circulating *Plasmodium* parasites. Hence, measures should be taken to evaluate the effectiveness of existing interventions. The population should be properly educated on malaria prevention in general and the proper use of LLIN, even beyond its intended efficacy life span, until replacement. The proportion of circumsporozoite protein positive mosquitoes in the sample was 34.2%, with an EIR of 0.808ib/P/n. Thus, unprotected individuals living in Minkoameyos during the study period were at the risk of receiving 0.808 infectious mosquito bites per night and consequently 294.92 infectious bites per year. The abundance and high infection status of *An. gambiae* and *An. funestus* confirm their role as the major malaria vectors in Cameroon, particularily in peri-urban areas [45, 48]. The roles of *An. ziemanni* and *An. paludis* as secondary malaria vectors, due to their minimal contribution in malaria transmission in localized areas were also confirmed. The two vectors can, therefore, contribute to maintaining transmission even on a small scale over a long period of time in this locality. However, due to their low density in this study, their actual contribution to malaria transmission needs to be investigated further [49]. None of the *An. brohieri* was positive for CSP. This species, known to be essentially zoophilic and rarely biting humans might, therefore, be suggested to have no role in malaria transmission in this locality [50].

### Effect of house improvement on entomological indices

The effectiveness of screening homes in reducing malaria incidence has been demonstrated in several studies in sub-Saharan Africa [29, 51]. Limiting vector entrance into the houses [52] could reduce the number of mosquito bites that could otherwise be infectious and consequently, indoor transmission [26]. *Anopheles* g*ambiae*, one of the most prevalent and important vectors of malaria in sub-Saharan Africa, constituted 95.2% of the total *Anopheles* species collected. The findings show that appropriate modification of houses can lead to a significant decrease in the indoor density of malaria vectors and the risk of exposure during the main vector feeding hours of the day by up to 50%. Higher reduction rates have been reported in several areas such as the Gambia, where improving houses through installation of insect-screen ceiling reduced house entry of *An. gambiae* mosquitoes by about 65% and 80% in 1987 and 2003, respectively [20, 26]. In southern Mozambique, covering gable end of houses with either untreated mosquito netting, shade clothes and deltamethrin-impregnated shade clothes reduced house entry of *An. gambiae* by 84%, 69% and 76%, respectively [53]. In a rice irrigation scheme area in lowlands of western Kenya, papyrus mats ceiling modification reduced house entry of *An. gambiae s.l.* and *An. funestus* densities by 78 to 80% and 86%, respectively compared to unmodified houses [52].

When comparing the night biting cycle of the indoor and outdoor mosquitoes, there was a significant reduction in mosquito abundance during the night, especially between 10 p.m. to 06 a.m.. This could be because *An. gambiae* is well adapted for entering houses through the eaves, since it flies upwards when encountering a vertical surface [39]. The peak indoor biting rates observed at 6:00 a.m. in improved houses and at 4:00 a.m. in non improved houses are not consistent with what has already reported [38, 46, 55]. The housing improvements implemented became significant barriers to mosquito entry into the house during their feeding times and during human resting time indoors. The difference observed in the indoor hourly variation of mosquitoes abundance between improved and non improved houses suggest that housing improvement may lead to a change in mosquitoes behaviour.

The relative number of indoor parous *An. gambiae* reduced significantly by 1.8-fold in improved houses. The indoor density of infected *Anopheles* mosquitoes (all species) also reduced by 1.8-fold in improved homes. These results highlight the trends and correlation between improved housing and the decrease in risk of exposure to malaria-carrying vectors. Infection rates and EIR were also lower in intervention houses; this may be due to factors such as household environment, and population knowledge, living and treatment-seeking habits. Housing improvements shielding home residents from exposure to and contact with potentially infected vectors have been shown to be a highly acceptable strategy often welcomed by the communities and households receiving it [54]. The additional comfort, improved aesthetics and noticeable relief from vectors could be the reason for such level of acceptance. The good uptake of this vector-control strategy indicates that there is important potential to scale-up similar interventions elsewhere in places of need. This study highlights the need for integrated approach to malaria control and further research on the effect of house improvement on malaria incidence rates, while controlling for other factors mentioned above.

This study presents certain limits. It did not account for socio-economic determinants of health, such as wealth and the possession of bed nets, which may considerably impact the number of vectors in the catches. Furthermore, the study did not control for LLIN position. The fact that the investigators were not blinded, and knew who slept in an improved house could be a source of bias and should be considered in subsequent studies. Despite these limits, the study provides useful baseline information that can be further exploited for improved malaria vector control in rural endemic settings. Thus, some relevant and important conclusions as well as significant trends can be drawn from this study.

## Conclusion

The screening and repairs made to the houses contributed to reducing the entry of malaria vectors into houses, thereby reducing human vector contact inside houses. This study conducted in a semi-urban area of Cameroon, with perennial malaria transmission. It therefore highlights the need for policy specialists to further evaluate and promote aspects of house design as a complementary control tool that could limit mosquitoes entering houses, thereby reducing human–vector contact and malaria transmission in endemic areas of similar settings. However, the efficacy of such intervention should be closely evaluated through larger studies including entomological, socio-anthropological and parasitological data collection.

## Data Availability

The datasets used and/or analysed during the current study are available from the corresponding author on reasonable request.

## Abbreviations

ARCHIVE: Architecture for Health in vulnerable environment
b/p/n: Bites per person per night
BTC: Biotechnology centre
CSP: Circumsporozoite protein
DNA: Deoxy ribonucleic acid
EIR: Entomological inoculation rate
ELISA: Enzyme-linked-immunosorbent assay
HLC: Human landing catch
ib/p/n: Infective bites per person per night
IE: Intervention effect
IR: Infection rate
LLIN: Long-lasting insecticide-treated nets
m.a: Man biting rate
n/a: Not applicable
NaOH: Sodium hydroxide
NMCP: National malaria control programme
PCR: Polymerase chain reaction
RF: Reduction factor
RFI: Reduction factor in improved houses
RFN: Reduction factor in non-improved houses
*s.l.*: Sensu lato
*s.s*: Sensu *stricto*
WHO: World Health Organization
